# Predicting respiratory failure in patients infected by SARS-CoV-2 by admission sex-specific biomarkers

**DOI:** 10.1186/s13293-021-00407-x

**Published:** 2021-11-22

**Authors:** Maria Teresa Pagano, Daniela Peruzzu, Luca Busani, Marina Pierdominici, Anna Ruggieri, Andrea Antinori, Gianpiero D’Offizi, Nicola Petrosillo, Fabrizio Palmieri, Pierluca Piselli, Stefania Cicalini, Stefania Notari, Emanuele Nicastri, Chiara Agrati, Giuseppe Ippolito, Francesco Vaia, Maria Cristina Gagliardi, Maria Rosaria Capobianchi, Elena Ortona, INMI-ISS COVID-19 team, Manuela Macchione, Rachele Di Lorenzo, Marta Camici, Roberta Gagliardini, Serena Vita, Gaetano Maffongelli, Eugenia Milozzi, Francesca Faraglia, Carlotta Cerva, Silvia Mosti, Davide Roberto Donno, Pierangelo Chinello, Veronica Bordoni, Alessandra Sacchi, Eleonora Tartaglia, Rita Casetti, Germana Grassi, Eleonora Cimini, Maria Luisa Dupuis, Simona Anticoli, Katia Fecchi, Maria Bellenghi, Rossella Puglisi, Gianfranco Mattia, Giada Pontecorvi

**Affiliations:** 1 Centro di Riferimento per la Medicina di Genere, Istituto Superiore di Sanità, Viale Regina Elena 299, 00161 Rome, Italy; 2 Istituto Nazionale per le Malattie Infettive Lazzaro Spallanzani (IRCCS), Via Portuense 292, Rome, Italy

**Keywords:** Biomarkers, Sex, Gender, COVID-19, Testosterone, Estrogen, Angiotensin1-7

## Abstract

**Background:**

Several biomarkers have been identified to predict the outcome of COVID-19 severity, but few data are available regarding sex differences in their predictive role. Aim of this study was to identify sex-specific biomarkers of severity and progression of acute respiratory distress syndrome (ARDS) in COVID-19.

**Methods:**

Plasma levels of sex hormones (testosterone and 17β-estradiol), sex-hormone dependent circulating molecules (ACE2 and Angiotensin1-7) and other known biomarkers for COVID-19 severity were measured in male and female COVID-19 patients at admission to hospital. The association of plasma biomarker levels with ARDS severity at admission and with the occurrence of respiratory deterioration during hospitalization was analysed in aggregated and sex disaggregated form.

**Results:**

Our data show that some biomarkers could be predictive both for males and female patients and others only for one sex. Angiotensin1-7 plasma levels and neutrophil count predicted the outcome of ARDS only in females, whereas testosterone plasma levels and lymphocytes counts only in males.

**Conclusions:**

Sex is a biological variable affecting the choice of the correct biomarker that might predict worsening of COVID-19 to severe respiratory failure. The definition of sex specific biomarkers can be useful to alert patients to be safely discharged versus those who need respiratory monitoring.

## Highlights

Sex should be considered as a biological variable influencing the choice of the appropriate predictive biomarker for lung failure in COVID-19.

Testosterone plasma levels were significantly higher in male patients with mild /no ARDS in comparison to those with moderate/severe ARDS.

Estrogen plasma levels correlated positively in COVID-19 female patients and correlated negatively in COVID-19 male patients with lung functionality.

Angiotensin1-7 plasma levels and neutrophil counts predicted the outcome of ARDS only in female patients, whereas testosterone plasma levels and lymphocytes count only in men.

## Background

Men and women are infected by SARS-CoV-2 at same rate, but men have shown higher risk of developing severe disease and more often undergo complications, including acute respiratory distress syndrome (ARDS), intensive care unit admission and death, compared to women [1–8]. However, factors determining the sex differences in COVID-19 have not been completely clarified yet. Several markers and risk factors for COVID-19 severity have been identified, such as D-Dimer and ferritin plasma levels, neutrophil and lymphocyte counts and the presence of comorbidities, but their link to sex-specific outcome of disease severity and lung disability has been scarcely investigated [9, 10]. Sex hormones, in particular estrogen and testosterone, have been suggested to play a crucial role in determining COVID-19 progression. Regarding testosterone, growing evidence suggests that the levels of this hormone are involved in disease progression [11, 12]. Testosterone has an anti-inflammatory effect, decreasing pro-inflammatory cytokines such as, IL-1β, IL-6, and TNF-α which have a central role in the progression of COVID-19 infection [13]. Accordingly, in elderly men the abating of testosterone correlates with the increase of a pro-inflammatory state [14]. Notably, plasma testosterone levels decrease in the presence of obesity and diabetes [15], which are comorbidities frequently associated to COVID-19. Interestingly, in male patients with COVID-19, low testosterone concentrations associated with increased disease severity and inflammation, suggesting a protective role for this hormone [16]. In contrast, other studies supported a pathogenic effect of testosterone, describing that androgen receptor activation increases the expression of the SARS-CoV-2 co-receptor transmembrane protease serine 2 (TMPRSS2) [11, 17]. Accordingly, a recent study showed that prostate cancer patients receiving androgen-deprivation therapies are partially protected from SARS-CoV-2 infections [18].

Estrogen, in particular 17β-estradiol (E2), has been observed to have a protective effect in COVID-19 hampering the inflammatory storm, promoting effective immune responses, and promoting the fusion of endosomes and lysosomes inducing the virus’ degradation [19]. Moreover, E2 is able to induce the expression of the angiotensin-converting enzyme 2 (ACE2) [20]. In addition to acting as SARS-CoV-2 receptor, ACE2 hydrolyses Angiotensin-II to Angiotensin-(1-7) (Ang1-7) which, by its anti-inflammatory effect, displays a protective role in several pathologies, such as hypertension, cardiovascular diseases, and ARDS that represent a risk of worse prognosis in COVID-19 [21–24]. According to ACE2 pulmonary protective effect, in animal models of acute lung injury, ACE2-knockout mice show a more severe lung failure compared with wild-type control mice [25]. Further supporting E2 protective effects, a positive association between COVID-19 and menopausal status has been detected [26, 27]. In addition, E2 therapy has been shown to reduce the fatality risk by more than 50% in female COVID-19 patients > 50 years [28]. In line with these data, lower rates of hospitalization, less need of respiratory support, and shorter period of hospitalization have been observed in pre-menopausal women compared to post-menopausal women [29]. Several clinical trial are underway testing the effect of E2 and estrogen receptor selective modulators on clinical response and mortality of COVID-19 (https://clinicaltrials.gov/).

Here, to the aim to identify sex-specific markers of severity and/or progression of COVID-19 we examined plasma levels of testosterone, E2, soluble ACE2 (sACE2) and Ang1-7 together with the known biomarkers for COVID-19 severity (D-Dimer and ferritin plasma levels, neutrophil and lymphocyte number) and the presence of pre-existing comorbidities in male and female patients taking into account the severity of respiratory disease at the time of admission and the occurrence of respiratory deterioration during hospitalization.

## Patients and methods

### Patients

160 adult patients (80 females and 80 age matched males) admitted to the National Institute for Infectious Diseases Lazzaro Spallanzani Hospital, Rome, Italy between March and September 2020, positive for SARS-CoV-2 by RT–PCR from nasopharyngeal swabs, and able to provide informed consent were enrolled. Intersex and transgender individuals were not represented in this study. Demographic and medical history, the presence of pre-existing comorbidities (diabetes, cancer, cardiological diseases, hypertension, asthma, respiratory diseases, kidney failure, liver failure, neurological disorders, metabolic disorders, obesity), and laboratory biomarkers (D-Dimer and ferritin plasma levels, neutrophil and lymphocyte number), were obtained and recorded for each patient upon admission to hospital. Laboratory biomarker analyses were repeated during hospitalization. Plasma samples were collected at clinical admission.

The arterial oxygen partial pressure (PaO_2_) to fractional inspired oxygen (FiO_2_) ratio (*P*/*F*), was assessed in each patient at admission and during hospitalization. The *P*/*F* cutoff assessment of 200 mmHg was used to distinguish two categories of patients: those with a *P*/*F* < 200 mmHg indicating moderate/severe ARDS [30, 31] and those with a *P*/*F* ≥ 200 mmHg, indicating mild/no ARDS. We divided patients in two further groups: (i) patients with deteriorated disease, when the worst *P*/*F* during hospitalization was < 200 mmHg, and was lower than that observed at admission, and (ii) patients with stabilized disease, when the worst *P*/*F* during hospitalization was *P*/*F* ≥ 200 mmHg, indicating mild/no ARDS, and when the worst *P*/*F* was < 200 mmHg**,** but higher or equal to that observed at admission.

The study was approved by our local ethics committee, and was conducted according to the Declaration of Helsinki. The patients provided their written informed consent to participate in this study.

### Measurement of plasma levels of testosterone, 17β-estradiol, sACE2 and Ang1-7

Plasma samples collected at admission were analysed by the following ELISA kits: Free Testosterone (R&D Systems, Minneapolis, MN, USA; Intra-Assay: CV 3.1%, Inter-Assay: CV 6.3%), 17β-estradiol (Abcam, Cambridge, UK; Intra-Assay: CV < 9%, Inter-Assay CV < 10%), sACE2 (Abcam, Intra-Assay: CV 2.3%, Inter-Assay: CV 3.2%) and Ang1-7 (Finetest, Wuhan, Hubei, China; Intra-Assay: CV < 8%, Inter-Assay: CV < 10%). All the ELISA kits were used according to manufactures 'instructions. No significant cross-reactivity or interference between Ang1-7 and analogues or other factors present in biological samples was observed.

### Statistical analysis

Data were summarized according to groups as medians and interquartile interval (25th–75th percentile), and percentages. Categorical variables, whenever dichotomous or nominal, were reported as frequencies and percentages and analysed through the Chi-square test. Statistical analysis for groups comparison was performed by the Mann–Whitney *U* test, Spearman’s rank correlation or the Chi-Square test or Fisher test as appropriate. A *P* value ≤ 0.05 was considered statistically significant.

The age, for privacy reason was not collected, only two age classes were defined, up to 45 years and 55 years and more, and the patients were assigned accordingly. No patients between 46 and 54 years were included.

Multivariable linear regression analysis was performed to assess independent relationships of *P*/*F* (in mmHg), age and the other biomarkers as predictors in women and men with COVID-19. For repeated measures, such lymphocytes and neutrophils count, the worst result was considered (lower lymphocyte count and higher neutrophils count). To summarize comorbidities was created a categorical variable with the following values: 0 = no comorbidities, 1 = one comorbidity, 2 = two or more comorbidities.

Variables from the univariate analysis that showed a significant correlation with the outcome were included in the multivariable linear regression analysis. The regression was stratified by sex to provide separate models for women and men.

STATA 16 (StataCorp, Texas USA) was used for statistical analysis.

## Results

### Analysis of circulating parameters in male and female COVID-19 patients divided according to *P*/*F* at hospital admission

Lymphocyte and neutrophil number, D-Dimer, ferritin, E2, testosterone, soluble ACE2 and Ang1-7 plasma levels were measured in total sex-aggregated patients and in female and male patients, stratified according to their *P*/*F* values at admission: *P*/*F* < 200 mmHg indicating moderate/severe ARDS and P/F ≥ 200 mmHg, indicating mild/no ARDS.

**Fig. 1:**
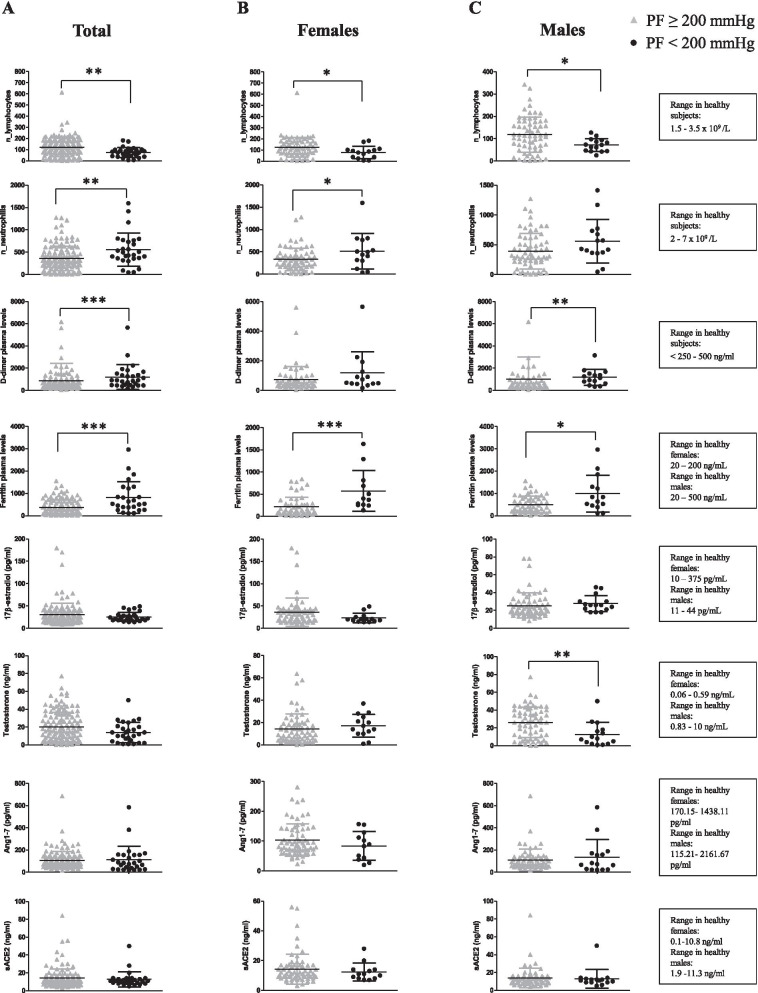
Analysis of circulating parameters in COVID-19 patients with moderate/severe and mild/no ARDS. Lymphocyte and neutrophil number, D-Dimer, ferritin, E2, testosterone, soluble ACE2 and Ang1-7 plasma levels were measured in total (**A**), female (**B**) and male (**C**) patients at time of admission to hospital. Patients were divided according to ARDS occurrence at time of admission (*P*/*F* < 200 mmHg indicating moderate/severe ARDS and *P*/*F*  ≥ 200 mmHg, indicating mild/no ARDS). Data referred to parameters are reported as mean ± standard deviation (SD). Data were analyzed using Mann–Whitney *U* test. *P < 0.05 was considered statistically significant

As shown in Fig. 1, lymphocyte number was significantly lower in patients with moderate/severe ARDS in comparison with patients with mild/no ARDS, sex independently. Neutrophil number was significantly higher in total and in female patients with moderate/severe ARDS than in those with mild/no ARDS, but not in male patients.

D-Dimer plasma level was significantly higher in total and male patients with moderate/severe ARDS. Interestingly, D-Dimer level did not vary in female patients according to *P*/*F* ratio, suggesting the usefulness of this biomarker only in males. Ferritin plasma level was significantly higher in patients with moderate/severe ARDS in comparison with patients with mild/no ARDS, both in female and male patients.

No significant differences were observed in plasma levels of E2, Ang1-7 and sACE2 in the two groups of patients stratified according to *P*/*F*, whereas testosterone level was significantly higher in plasma from male patients with mild/no ARDS than in those with moderate/ severe ARDS.

**Fig. 2:**
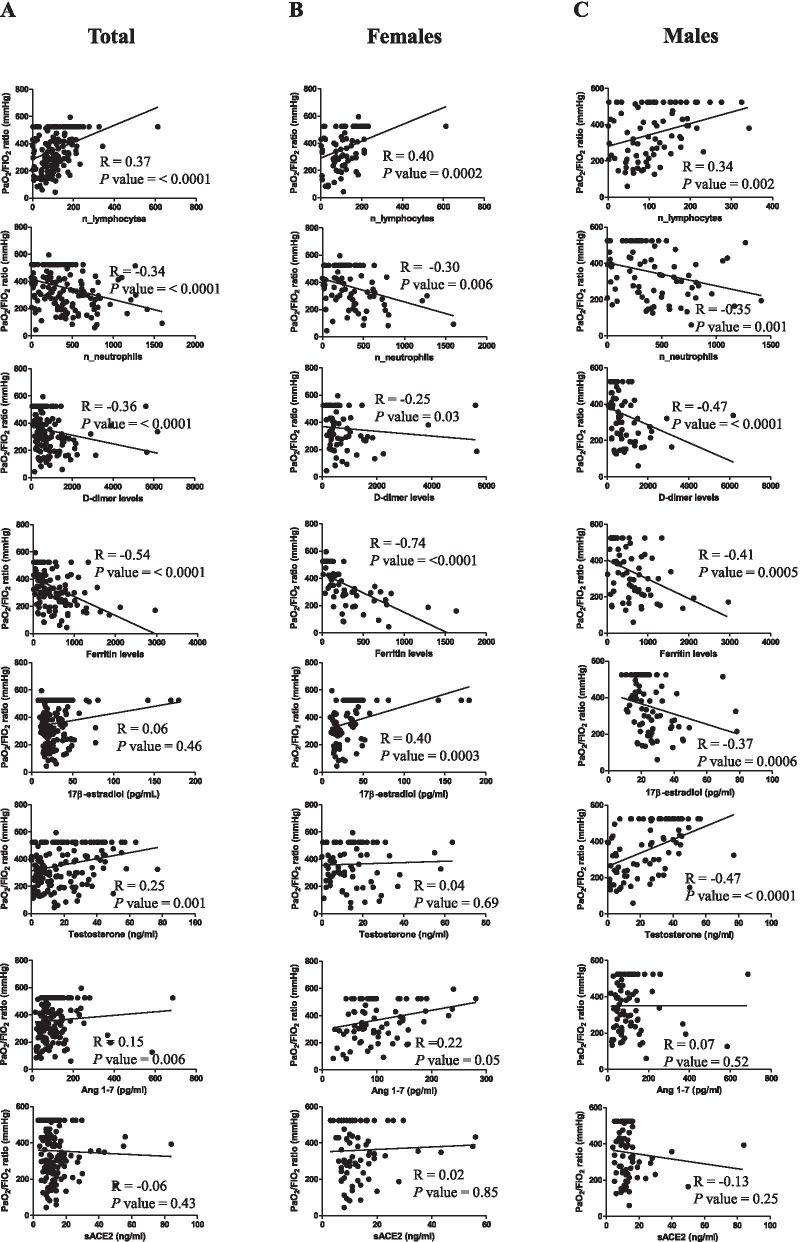
Correlation of *P*/*F* with circulating parameters in COVID-19 patients. Correlation and linear regression analysis of *P*/*F* with lymphocyte number, neutrophil number, D-Dimer, ferritin, E2, testosterone, soluble ACE2 and Ang1-7 plasma levels in total (**A**), female (**B**) and male (**C**) patients. The Spearman’s rho (*R*) and *P* values were determined using the Spearman’s rank correlation analysis. Solid lines represent best fits as estimated by linear regression analysis. *P < 0.05 was considered statistically significant

From the correlation analysis of *P*/*F* with the circulating parameters (Fig. 2) a significant positive correlation of lymphocyte number and a significant negative correlation of neutrophil number were found in total patients, as well as in female and male patients. *P*/*F* showed a significant negative correlation also with plasma D-dimer and ferritin. Regarding E2 levels, we observed no correlation with *P*/*F* in total patients, but, interestingly, we observed a significant positive correlation in female and a significant negative correlation in males. Testosterone levels positively correlated with *P*/*F* but only in male patients and Ang1-7 positively correlated only in females, supporting its indirect dependence by estrogens that induce ACE2 expression. No correlation was observed between *P*/*F* and sACE2.

### Analysis of circulating parameters in male and female COVID-19 patients divided according to deterioration or stabilization of lung disease during hospitalization

**Fig. 3:**
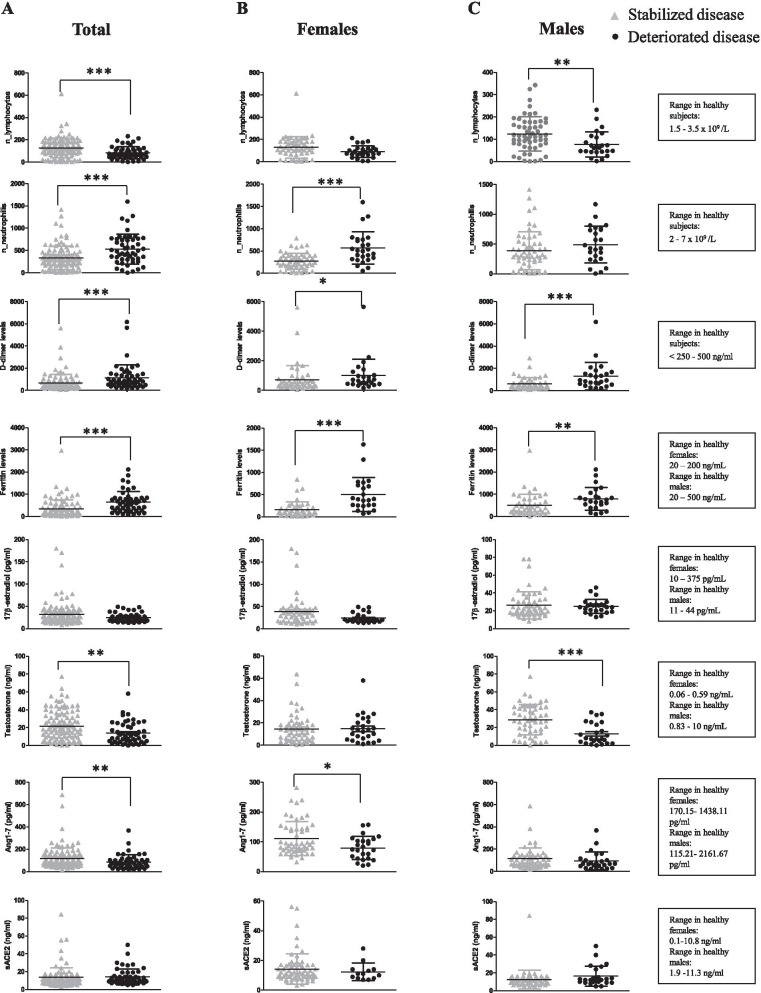
Analysis of circulating parameters in stabilized and deteriorated COVID-19 patients. Lymphocyte and neutrophil number, D-Dimer, ferritin, E2, testosterone, soluble ACE2 and Ang1-7 plasma levels were analysed in total (**A**), female (**B**) and male (**C**) patients, divided in two groups: (i) patients with deteriorated disease, when the worst *P*/*F* during hospitalization was < 200 mmHg and was lower than that observed at admission; (ii) patients with stabilized disease, when the worst *P*/*F* during hospitalization was *P*/*F*  ≥ 200 mmHg, indicating mild/no ARDS and when the worst *P*/*F* was < 200 mmHg**,** but higher or equal to that observed at admission. Data referred to parameters are reported as mean ± SD. Data were analysed using Mann–Whitney *U* test. *P < 0.05 was considered statistically significant

The circulating parameters reported above were also analysed in total, sex-aggregated patients, and in female and in male patients, divided in two groups according to the deterioration or stabilization of lung disease as described in “Patients and methods” (Fig. 3). Lymphocyte and neutrophil counts were significantly lower and higher, respectively, in total patients with deteriorated disease than in those with stabilized disease. To note, a significant association with disease deterioration was detected only in men for lymphocyte count and only in women for neutrophil count. These data support a sex-specific value of these biomarkers to predict disease progression.

D-Dimer and ferritin plasma levels were significantly higher in total, male and female patients with deteriorated disease than in those with stabilized disease.

No significant differences were observed in E2, and sACE2 plasma levels, whereas testosterone and Ang1-7 were significantly lower in total patients with deteriorated disease than in those with stabilized disease. Disaggregating by sex, testosterone and Ang1-7 were significantly lower in male and in female patients with deteriorated disease, respectively, than in those with stabilized disease.

### Pre-existing co-morbidities in male and female COVID-19 patients stratified according to *P*/*F*

The percentage of patients with moderate/severe ARDS during hospitalization and with pre-existing diabetes, cancer, cardiological diseases, hypertension, respiratory diseases and metabolic diseases was significantly higher than the percentage of patients with the same comorbidities but with mild/no ARDS (Table 1). Asthma, liver and kidney failure, neurological diseases and obesity did not show any association with ARDS severity in total, sex-aggregated patients. Female patients with pre-existing diabetes, tumor, cardiological diseases, hypertension, metabolic diseases and obesity had more frequently moderate/severe ARDS during hospitalization than mild/ no ARDS. In male patients, hypertension, and metabolic diseases were significantly associated with moderate/severe ARDS at time of admission.

**Table 1:** Pre-existing comorbidities in total, male and female patients with COVID-19 divided according to the *P/F* ratio during hospitalization (*P/F* < 200 mm Hg and *P/F* ≥ 200 mm Hg)

Comorbidities			*P/F* < 200 mmHg (n. 55)	*P/F* ≥ 200 mmHg (n.105)	*P* value by Chi-Square test
Diabetes	Total (n.18)		11	7	**0.017**
	Females (n. 6)		5	1	**0.013**
	Males (n. 12)		6	6	0,336
Tumor	Total (n.11)		9	2	**0.001**
	Females (n.6)		5	1	**0.013**
	Males (n.5)		4	1	0.055
Cardiological diseases	Total (n.33)		19	14	**0.003**
	Females (n.16)		10	6	**0.007**
	Males (n.17)		9	8	0.155
Hypertension	Total (n.51)		31	20	** < 0.001**
	Females (n.20)		14	6	** < 0.001**
	Males (n.31)		17	14	**0.009**
Asthma	Total (n.5)		1	4	0.661
	Females (n.4)		1	3	1.000
	Males (n.1)		0	1	1.000
Respiratory diseases	Total (n.15)		9	6	**0.043**
	Females (n.4)		2	2	0.592
	Males (n.11)		7	4	0.087
Kidney failure	Total (n.2)		2	0	0.117
	Females (n.1)		1	0	0.325
	Males (n.1)		1	0	0.362
Liver failure	Total (n.6)		2	4	1.000
	Females (n.2)		1	1	0.547
	Males (n.4)		1	3	1.000
Neurological diseases	Total (n.11)		6	5	0.189
	Females (n.8)		5	3	0.105
	Males (n.3)		1	2	1.000
Metabolic disorders	Total (n.29)		20	9	** < 0.001**
	Females (n.17)		12	5	** < 0.001**
	Males (n.12)		8	4	**0.024**
Obesity	Total (n.19)		9	10	0.210
	Females (n.11)		7	4	0.033
	Males (n.8)		2	6	0.704
One comorbidity	Total (n.111)		51	60	** < 0.001**
	Females (n.54)		25	29	** < 0.001**
	Males (n.57)		26	31	**0.009**
Two or more comorbities	Total (n.51)		33	18	**0.000**
	Females (n.24)		16	8	**0.000**
	Males (n.27)		17	10	**0.0006**

Bold values indicate statistical significance at the* p* ≤ 0.05 level

### Associations between the analysed parameters in female and in male COVID-19 patients separately by multivariate linear regression analyses

Multivariate linear regression analyses were performed separately by sex (Table 2). The model incorporating age classes (up to 45 years and 55 years and more) and comorbidities, revealed that in women, being 55 or more years old (*β* = − 72.061; 95%CI from − 131.901 to − 12.221), having a high number of neutrophils (*β* = − 8.575; 95%CI from − 12.806 to − 4.344) and high ferritin concentration (*β* = − 0.032; 95%CI from − 0.060 to − 0.005) was negatively associated with *P/F*. In women a positive association with *P/F* was observed for E2 concentration (*β* = 0.766; 95%CI from 0.017 to 1.514) and lowest lymphocyte count (*β* = 0.451; 95%CI from 0.097 to 0.805).

**Table 2:** Multivariable linear regression models to assess independent relationships of *P/F* (in mmHg), age and the other biomarkers as predictors in women and men with COVID-19

Predictors	Dependent variable: *P/F* (mm Hg)							
	Women				Men			
	Adjusted coefficient	95% Conf. Int		*P*	Adjusted coefficient	95% Conf. Int		*P*
Age class (≤ 45 vs 55 ≥)	− 72.061	− 131.901	− 12.221	0.019	− 56.563	− 116.151	3.024	0.062
Number of lymphocytes in the lowest count (n. cells/L)	0.451	0.097	0.805	0.014	0.242	− 0.169	0.653	0.243
Number of neutrophils in the highest count (n. cells/L)	− 8.575	− 12.806	− 4.344	< 0.001	− 4.222	− 6.693	− 1.751	0.001
Ferritin concentration (ng/mL)	− 0.032	− 0.060	− 0.005	0.020	− 0.069	− 0.113	− 0.025	0.003
17β-estradiol concentration (pg/mL)	0.766	0.017	1.514	0.045	− 1.098	− 2.756	0.561	0.191
Testosterone concentration (ng/mL)	− 0.371	− 2.243	1.500	0.692	1.385	0.002	2.767	0.050
Comorbidities								
No comorbidities	1 (ref.)				1 (ref.)			
One comorbidity	− 21.540	− 81.161	38.081	0.472	− 44.305	− 94.975	6.366	0.085
Two or more comorbidities	− 35.351	− 105.264	34.561	0.316	− 66.145	− 125.909	− 6.381	0.031

Men showed that having two or more comorbidities (*β* = − 66.145; 95%CI from − 125.909 to − 6.381), a high number of neutrophils (*β* = − 4.222; 95%CI from − 6.693 to − 1.751) and high ferritin concentration (*β* = − 0.069; 95%CI from − 0.113 to − 0.025) was negatively associated with *P*/*F*, while positive association was observed with testosterone concentration (*β* = 1.385; 95%CI from − 0.002to 2.767).

## Discussion

COVID-19 causes a wide range of disease, from asymptomatic to severe respiratory failure. After SARS-CoV-2 infection some patients develop a rapidly progressive respiratory failure that requires ventilatory support. Several biomarkers have been identified to predict the outcome of COVID-19 severity [32–34] but few data are available on sex difference in their predictive value and in risk factors for COVID-19 progression [10, 35]. Since sex differences in COVID-19 lethality rate exist, it is conceivable to hypothesize that some biomarkers could predict disease severity in a sex specific manner. Accordingly, experimental and epidemiologic evidences suggest that most of the biomarkers that have been tested in the context of the risk of infection and the severity of COVID-19 are different in healthy male and female [36]. To clarify this point, we analysed plasma levels of sex hormones, and sex-hormone dependent circulating molecules, i.e., sACE2 and Ang1-7 together with the known biomarkers for COVID-19 severity (D-Dimer and ferritin plasma levels) and with the occurrence of respiratory deterioration during hospitalization and we observed that some biomarkers could be useful both in males and in females but others are sex specific (Table 3). We also evaluated whether the presence of pre-existing comorbidities was associated with ARDS severity in a sex-dependent manner.

**Table 3:** Correlation of the sex-specific biomarkers and the acute respiratory distress syndrome (ARDS) stage in the COVID-19 patients

Admission biomarkers for female patients	
Moderate / severe ARDS (at admission)	Deteriorated ARDS (during hospitalization)
↓ Lymphocytes counts	**↑ Neutrophil counts**
**↑ Neutrophil counts**	↑ D-Dimer plasma levels
↑ Ferritin plasma levels	↑ Ferritin plasma levels
	**↓ Ang1-7 plasma levels**

Admission biomarkers for male patients	
Moderate / severe ARDS (at admission)	Deteriorated ARDS (during hospitalization)
↓ Lymphocytes counts	**↓ Lymphocytes counts**
↑ Ferritin plasma levels	↑ D-Dimer plasma levels
**↑ D-Dimer plasma levels**	↑ Ferritin plasma levels
**↓ Testosterone plasma levels**	**↓ Testosterone plasma levels**

Biomarkers specific only for female or male patients are indicated in bold

Considering already known circulating biomarkers, we found that ferritin levels were significantly higher and lymphocyte count significantly lower, at admission, in patients with moderate/severe ARDS in comparison to those with no or mild ARDS both by sex-aggregated and by sex-disaggregated analyses, confirming data present in literature regarding the role for these circulating parameters as biomarkers of COVID-19 severity [10, 37, 38].

With regard to neutrophil count and D-Dimer level, our data showed that their plasma levels significantly increased in patients with moderate/severe ARDS in comparison to those with no or mild ARDS when sex-aggregated analysis was performed, confirming previous reported data [12, 37–39]. Disaggregating the data by sex, the increased neutrophil count was observed only in females, whereas the increased D-Dimer level only in males both with moderate/severe ARDS, supporting the crucial value of considering sex differences in the search of clinical biomarkers in COVID-19.

With regard to the role of these circulating parameters as predictive biomarkers, all of them had a significant value in predicting worsening of the disease during hospitalization in total patients. However, sex disaggregated analysis of the data suggested a sex-linked role of the circulating biomarkers in worsening the outcome of COVID-19 in the hospitalized patients. In fact, we found that, whereas D-Dimer and ferritin were useful predictors of deterioration both in male and in female patients, lymphocyte count were significantly lower in men with a deteriorated disease than in those with a stabilized disease. At opposite, neutrophil count increased only in women with a deteriorated disease than in those with a stabilized one.

We also found that plasma levels of testosterone were significantly higher in male patients with mild /no ARDS in comparison to those with moderate/severe ARDS and in male patients with stabilized disease then in those with a deteriorated disease. Accordingly, a randomized controlled trial described a progress in respiratory functionality in men taking testosterone replacement therapy [40]. Moreover, a decline in testosterone has been demonstrated to be predictive of a poor prognosis in men with COVID-19 [41]. Recently, a low level of testosterone was found to be a marker of clinical severity of COVID-19 in men [42] and lower testosterone concentrations during hospitalization were associated with increased disease severity and inflammation in men [16]. Hence, these data supported a protective role of testosterone and the usefulness of measuring its plasma level to predict COVID-19 progression in men. As regard to estrogen, we observed that E2 and pulmonary functionality positively correlated in women and negatively correlated in men. This sex-dependent opposite effect could be explained by the biphasic effect of E2 depending on its concentration. At high levels, e.g., periovulatory to pregnancy levels, E2 has mainly anti-inflammatory effects, by inhibiting the production of pro-inflammatory cytokines, such as TNF-α, IL-1β and IL-6, involved in cytokine storm and disease severity [43, 44]. At low levels, as those observed in plasma from male patients, E2 stimulates the production of pro-inflammatory cytokines.

To note, E2 is able to upregulate the expression of ACE2 [20]. Furthermore, the gene encoding ACE2 is located on the X-chromosome, in sites commonly escaping the inactivation of one X-chromosome in mammalian XX cells (XCI), supporting the higher expression of this receptor in female than in males [24, 45]. During infection, binding of viral surface spike (S) glycoprotein to membrane-bound ACE2 (mACE2) triggers ACE2 shedding. The shed soluble ACE2 (sACE2) retains its catalytic activity, but its precise role in viral entry is still unclear. Several studies have reported the beneficial and preventive role of therapeutic sACE2 in COVID-19 [46–49]. Surprisingly, clinical data suggest that patients with low mACE2 and high sACE2 faced more disease severity and fatality [49, 50]. In this study, in total patients and also disaggregating by sex, in female and in male patients, we did not observe a significant association of sACE2 levels neither with ARDS severity at admission time nor with deterioration of disease during hospitalization, denying a possible use of this molecule as a biomarker of lung failure.

Regarding Ang1-7, this molecule is indirectly modulated by E2 which upregulates ACE2 expression. Accordingly, we observed that Ang1-7 positively correlated with P/F and its decrease predicted disease deterioration in total patients, but disaggregating data by sex showed that its predictive value was valid only for female and not for male patients.

With regard to the role of pre-existing comorbidities as sex-specific risk factors of ARDS progression, the pre-existence of hypertension and metabolic diseases were associated with a more severe disease in males, while pre-existing diabetes, tumors, cardiological diseases, hypertension, metabolic diseases and obesity appeared to be risk factors for more severe ARDS in females.

To explore the associations between all the analysed parameters in female and in male patients separately, we applied multivariate linear regression models and we observed that, considering age, women, being 55 or more years old, having a high number of neutrophils, and high ferritin concentration were associated with worst lung functionality, whereas E2 concentration was associated with better pulmonary function. In men, the presence of two or more comorbidities, a high number of neutrophils and high ferritin concentration negatively impacted on lung functionality, while high testosterone concentration was associated with better pulmonary function.

A limitation that could have reduced the capacity of our study to better discriminate between the severity of the disease and the sex related conditions was the focus on a single COVID-19 associated parameter, the *P/F* ratio only, without taking into account the final outcome. Another limitation was the categorization in two age groups only, due to restriction to the individual data for privacy reasons that, although allowed to divide according to the menopause status (mainly for women), did not allow a finer age-related analysis of the correlation.

## Perspectives and significance

Our data, although requiring validation through a wider population analysis, highlight that sex should be considered as biological variable influencing the choice of the appropriate biomarker and underline the need to personalize the assistance of patients from the time of admission also basing on sex.

## Data Availability

All data generated or analyzed during this study are included in this published article. Raw data may be provided via direct contact with the corresponding author.
